# Influence of hydrolyzable vs. condensed tannins supplementation in Liaoning cashmere goats: growth performance, nitrogen metabolism, rumen fermentation, and blood metabolites

**DOI:** 10.5713/ab.25.0367

**Published:** 2025-09-30

**Authors:** Xin Zhu, Di Han, Lu Zhang, Tingting Su, Lisha Ye, Haiying Liu, Xingtang Dou

**Affiliations:** 1 College of Animal Science and Veterinary Medicine, Shenyang Agricultural University, Shenyang, China; 2 Liaoning Agricultural and Rural Development Service Center, Shenyang, China; 3 State Key Laboratory of Animal Nutrition, College of Animal Science and Technology, China Agricultural University, Beijing, China; 4 Liaoning Cashmere Goat Breeding Farm Co., Ltd., Liaoyang, China

**Keywords:** Goat, Growth Performance, N Metabolism, Rumen Fermentation, Tannin

## Abstract

**Objective:**

This study was conducted to investigate the effects of hydrolyzable versus condensed tannins on growth performance, nitrogen (N) metabolism, rumen fermentation, and blood parameters in Liaoning cashmere goats.

**Methods:**

A control diet and three experimental diets—supplemented with 0.5% chestnut tannin (CT), tannic acid (TA), or quebracho tannin (QT)—were compared.

**Results:**

Goats consuming the QT diet had higher average daily gain and average daily feed intake compared to those on the TA diet (p<0.05). Compared to the control group, the QT group exhibited lower fecal N, urine N, and total N output (g/d, p<0.05), as well as higher N retention (g/d) and N utilization (g/kg N intake) relative to the CT and TA groups (p<0.05). The QT group had lowest urine uric acid level, while the CT and TA groups showed lower creatinine levels compared to the control (p<0.05). Tannin supplementation increased rumen ammonia-N (NH_3_-N) concentration and reduced protozoa counts (p<0.05). Plasma concentrations of total protein, globulin, superoxide dismutase, catalase, and lysozyme were higher in the QT group than in the CT and TA groups (p< 0.05).

**Conclusion:**

These results suggest that condensed tannin provides greater advantages than hydrolyzable tannin on growth, N balance, and antioxidant function at equivalent dietary inclusion levels.

## INTRODUCTION

Nitrogen (N) utilization efficiency in ruminants is generally low, primarily dependent on N intake and excretion [1]. Up to 70% of ingested N is excreted as feces and urine in ruminants [2]. Higher N excretion not only increases nitrogenous pollutant emissions but also reduces protein deposition, impairing meat and milk production in ruminants. Moreover, excessive or high-quality dietary protein can be fermented by rumen microorganisms into ammonia, reducing protein bioavailability [3]. Therefore, protecting dietary protein from ruminal degradation is critical to improve N utilization.

Tannins, plant-derived polyphenols, form stable complexes with proteins via phenolic-carbonyl interactions, reducing ruminal degradation [4]. These complexes dissociate in the acidic abomasum and small intestine, enhancing post-ruminal protein availability and N utilization efficiency [5]. While high tannin levels impair nutrient digestibility and growth in monogastric animals [6], moderate supplementation in ruminants may improve feed efficiency by modulating rumen fermentation and microbial population and/or activity [7,8]. Depending on the source, molecular and chemical structure, and consumed amount, the effects of tannins on ruminants can be varied.

Hydrolyzable tannin and condensed tannin are two main groups of plant tannins. The former, with molecular weights ranging from 500 to 3,000 Daltons (Da), consists of polyphenol nuclei, whereas the latter, ranging from 1,000 to 20,000 Da, is oligomeric or polymeric flavonoids derived from flavane-3-ols such as catechin, epicatechin, gallocatechin, and epigallocatechin [9]. Chestnut tannin (CT) and quebracho tannin (QT) are the most widely available hydrolyzable and condensed tannins sources, respectively, which have been used in animal production [10]. Numerous studies have highlighted the beneficial effects of both hydrolyzable and condensed tannins in ruminants are related to their antimicrobial, antioxidant, and anti-inflammatory activities, thus contributing to improving animal health and growth performance [11–13]. Gao et al [14] reported that dietary CT supplementation enhanced the average daily gain (ADG) of finishing Tan lambs in a dose-dependent manner, modulated plasma metabolites and antioxidant capacity, and improved rumen fermentation. Costa et al [15] found that inclusion of condensed tannin above 40 g/kg in the diet had deleterious effects on feed intake, nutrient digestibility and N balance, thereby reducing the growth performance of the lambs, while another study found that feeding QT to sheep enhanced rumen fermentative activity [16]. Due to differences in functional moieties and bioactive properties, hydrolyzable and condensed tannins were previously demonstrated to show varied capacities to bind proteins and manipulate rumen fermentation, but the mechanism of action is unclear and should be studied further [17].

The Liaoning cashmere goat is famous for the highest cashmere yield, excellent cashmere quality, strong adaptability, and stable genetic performance [18]. While extensive research has focused on enhancing hair follicle development and improving cashmere production through nutritional interventions, strategies to optimize N metabolism and rumen fermentation in this breed remain underexplored [19]. To date, little research has been conducted on tannin supplementation in Liaoning cashmere goats. Our pervious study demonstrated that dietary supplementation with 0.5% hydrolyzable tannin (tannic acid, TA) or condensed tannin (QT) did not adversely affect feed intake and nutrient digestion but significantly modulated rumen microbiota composition, enhanced metabolite profiles, and reduced the abundance of methanogenic archaea (e.g., *Methanobrevibacter*) [20]. Based on these findings, we hypothesized that supplementation with hydrolyzable and condensed tannins would improve N utilization efficiency, rumen fermentation, and antioxidant capacity in Liaoning cashmere goats. Therefore, this study was conducted to evaluate the effect of dietary supplementation with hydrolyzable and condensed tannins on the growth performance, N metabolism, rumen fermentation, and blood parameters in Liaoning cashmere goats.

## MATERIALS AND METHODS

### Animals, diets, and management

Both CT (purity: ≥75%) and QT (purity: ≥73%) were provided by the Silvateam Trading. TA (purity: ≥95%) was purchased from the Henan Wanbang Chemical Technology.

Thirty-six female Liaoning cashmere goats with initial body weight (BW) of 32.59±2.15 kg and age of 18 months were selected and randomly divided into 4 groups (n = 9 goats/group). Each goat was housed in a single metabolism cage (1.2 m length×0.6 m width×1.6 m height), and had free access to feed and fresh water. All goats were healthy and free from parasites and diseases with a regular protocol of vaccines. A basal total mixed ration (control) was provided ad libitum for all goats during a 14-days adaptation period. The same content (0.5%) of tannins from different sources was added to the basal diet during the 42-days experimental period to formulate the experimental diets: 0.5% CT, 0.5% TA, and 0.5% QT, respectively. The basal diet was formulated according to the Nutrient Requirements of Cashmere Goats of China (NY/T 4048-2021) [21] and the ingredients and chemical composition are shown in Table 1.

### Sampling

During the experimental period, feed and orts were collected once daily at 08:00 h before feeding. Subsamples (10% of total collection) were taken per goat and pooled by replicate at the end of the experiment. The samples were ground to pass a 2-mm screen and were then used for chemical analysis. During the last week of the experimental period, urine was completely collected using a rubber funnel connected to a plastic tube and a plastic bucket containing a 10% sulphuric acid (H_2_SO_4_) solution to ensure that the pH<3. A 1% aliquot of total collection samples in each day were mixed by replicate and then stored at −20°C for chemical analysis. Daily total collection of feces from days 36 to 42 was carried out, and subsamples (10% of total collection) were mixed by replicate and then stored at −20°C for chemical analysis. On day 42, before feeding in the morning (08:00 h), 50 mL rumen fluid was orally collected from each goat by using a gastric tube, and after measuring pH and filtering though a 4-layer gauze, samples were stored at −20°C for further analysis. On the final day of the experiment, 10 mL blood was taken through the jugular vein using evacuated heparin sodium tubes from each goat before morning feeding. The blood was centrifuged at 2,000×g for 15 min to obtain plasma and stored at −20°C for later analysis.

### Laboratory analysis

The dry matter (DM, method 934.01) and ether extract (EE, method 920.39) of feed and fecal samples were determined according to AOAC International [22]. The neutral detergent fiber (NDF) and acid detergent fiber (ADF) were analyzed on an Ankom A200i Fiber Analyzer (ANKOM Technology). The N content of feeds, feces and urine samples was determined using the Kjeldahl method (method 968.06). The CP content was calculated as N×6.25.

The creatinine, uric acid, urea, and allantoin in urine samples were determined using commercial kits, which were provided by the Nanjing Jiancheng Bioengineering Institute.

The rumen fluid samples for volatile fatty acids (VFAs) analysis were centrifuged at 3,000×g for 15 min, and then 0.1 mL supernatant was mixed with 0.2 mL 25% metaphosphoric acid. After ice-water incubating for 30 min, the mixture was centrifuged at 10,000×g for 10 min to collect the supernatant. A 0.22 μm filter membrane was used to filter the supernatant and 1 μL of filtered fluid was injected into a gas chromatograph system (7890B, Agilent) equipped with a chromatographic column (30 m×0.25 mm×0.25 μm, FEAP, Dikma) to determine the concentrations of acetate, propionate, butyrate, isobutyrate, valerate, and isovalerate acids. The ammonia-N (NH_3_-N) concentration was determined following the method described by Weatherburn [23]. The microbial protein (MCP) concentration was determined as described by Makkar et al [24]. The number of ciliate protozoa was analyzed by using a counting chamber and a light microscope following the procedure described by Guimarães et al [25].

Biochemical (total protein, albumin, globulin, urea N, uric acid, aspertate aminotransferase [AST], alanine transaminase [ALT], total cholesterol, triglyceride, and blood glucose), antioxidant (superoxide dismutase [SOD], catalase [CAT], alkaline phosphatase [ALP], lactic dehydrogenase [LDH], malonaldehyde [MDA], and total antioxidant capacity [T-AOC]), and immune (immune globulin A [IgA], immune globulin G [IgG], immune globulin M [IgM], and lysozyme) indicators in the plasma were determined according to the guidelines of commercial kits (Sinoukbio).

### Calculations and statistical analysis

Daily feed intake and orts and weekly BW measurements were performed to calculate average daily feed intake (ADFI), ADG, and feed-to-gain ratio (F/G) within each replicate.

Apparent nutrient digestibility was calculated as nutrient digestibility (g/kg) = (feed nutrient intake – fecal nutrient output) / feed nutrient intake.

Parameters of N metabolism were included and calculated as follows: total digestible N (g/d) = feed N intake – fecal N output; N retention (g/d) = total digestible N (g/d) – urine N output (g/d). N utilization (g/g) = N retention (g/d) / feed N intake (g/d).

Data obtained from this study are presented as the mean± standard error and statistically analyzed using a one-way analysis of variance (ANOVA) procedure, and the Duncan’s multiple range test method was used to compare the significance of differences among means using SPSS 23.0 software (SPSS). Significant differences were declared when p≤0.05.

## RESULTS

### Growth performance

Table 2 shows that tannin addition had no effect on the BW and F/G (p>0.05). However, ADG in the QT group was higher than that in the control and TA groups from 1 to 21 d, 22 to 42 d, or 1 to 42 d (p<0.05), but did not differ from the CT group (p>0.05). Goats in the QT group had the highest ADFI, while those in the TA group had the lowest ADFI among groups from 1 to 21 d, 22 to 42 d, or 1 to 42 d (p<0.05).

### Nutrient digestibility

There were no significant differences in the DM, CP, EE, and NDF intake among groups (p>0.05), while the CT group had a higher ADF intake than the QT group (p<0.05), as shown in Table 3. Dietary supplementation with different tannins had no significant effects on the apparent digestibility of DM, EE, NDF, and ADF (p>0.05). However, the CP digestibility in the CT and TA groups was higher than in the control and QT groups (p<0.05).

### N metabolism

The results in Table 4 show that supplementing tannins affected fecal N output, urine N output, total N output, digestible N, N retention, and N utilization efficiency (p<0.05), but had no significant effects on N intake, ratio of fecal N output to total N output, and ratio of urine N output to total N output (p> 0.05). Compared to the control, the CT, TA, and QT groups had a lower fecal N output (p<0.05), the QT had a lower urine N output (p<0.05), and the CT and QT groups had a lower total N output (p<0.05). Tannin supplementation significantly increased amounts of daily digestible N and N retention, and N utilization efficiency (p<0.05). Among all groups, the QT had higher N retention and N utilization efficiency than the CT and TA groups (p<0.05), and the control had the lowest N retention and N utilization efficiency (p<0.05).

### Urine N constituents

As shown in Table 5, there was no difference in the output of urea among groups (p>0.05). The output of allantoin in the TA and QT groups was higher than that in the control and CT groups (p<0.05). Compared to the control, the output of uric acid in the CT and TA groups decreased (p<0.05), and the lowest output of uric acid was observed in the QT group (p<0.05). The output of creatinine in the CT and TA groups was lower than that in the control and QT groups (p<0.05).

### Rumen fermentation

Results from Table 6 showed that TA supplementation decreased rumen pH than the control, CT, and QT supplementation (p<0.05). Dietary supplementation with CT, TA, and QT increased (p<0.05) the concentration of NH_3_-N and decreased (p<0.05) the count of protozoa, but did not affect the concentration of MCP in the rumen (p>0.05). In addition, there was no significant difference in the concentration of total VFAs, acetate, propionate, butyrate, valerate, isovalerate acids and ratio of acetate to propionate acids (p>0.05), except that the CT group had a higher isobutyrate acid concentration than the TA group (p<0.05).

### Plasma indicators

As shown in Table 7, dietary supplementation with tannin did not affect the concentrations of plasma urea N, uric acid, AST, ALT, Triglyceride, and glucose (p>0.05), but affected the concentrations of total protein, albumin, globulin, and total cholesterol (p<0.05). Compared to the control, the CT and TA groups showed a higher concentration of total protein and globulin (p<0.05), and the QT showed the highest concentration of total protein and globulin (p<0.05). The TA and QT groups had a higher concentration of albumin compared to the control (p<0.05). In addition, the TA and QT groups had a higher concentration of total cholesterol than the control and CT groups (p<0.05).

Results from Table 8 present that there was no significant difference in the plasma concentration of ALP and LDH among groups (p>0.05). Compared to the control, the plasma concentration of SOD and CAT in the QT group increased (p<0.05), while that in the TA group decreased (p<0.05), but those indicators were not different between the CT and TA groups (p>0.05). Dietary supplementation with CT, TA, and QT decreased the concentration of MDA compared to the control (p<0.05), and the TA and QT groups showed a higher concentration of T-AOC than the control and CT groups (p<0.05).

There was no significant difference in the plasma concentration of IgG and IgM among groups (p>0.05), but the QT group showed an increased concentration of IgA than other groups (p<0.05). Compared to the control, all tannin groups increased the concentration of lysozyme (p<0.05), and the QT group had a higher concentration of lysozyme than the CT and TA groups (p<0.05) (Table 9).

## DISSCUSSION

Our results demonstrated that dietary supplementation with both hydrolyzable tannin and condensed tannin, at levels up to 0.5% of the diets, had no significant effects on BW or F/G in Liaoning cashmere goats. Notably, condensed tannin supplementation even increased feed intake and weight gain. Nevertheless, we reported here that hydrolyzable tannin, such as TA, has a great capacity to reduce feed intake and BW gain than condensed tannin, such as QT. A rapid decrease in palatability of feed due to the astringent taste of hydrolyzable tannin than condensed tannin may explain the reduction in feed intake in goats fed with hydrolyzable tannin diets. Hydrolyzable tannin can be hydrolyzed into gallic or ellagic acid, which are rich in phenolic hydroxyl groups and have good water solubility [26]. When goats were fed with hydrolyzable tannin, the astringent activity of tannin could be rapidly sensed by the gustatory system of animals, thus impairing voluntary feed intake and retarding weight gain. On the contrary, condensed tannin is non-hydrolyzable oligomeric and polymeric proanthocyanidins, consisting of coupled catechin units [27]. Because of varied degrees of polymerization, the spatially local concentration of phenolic hydroxyl groups per unit mass is relatively low in condensed tannin [28], which may have a reduced stimulatory effect on the taste sense of goats, thus leading to negligible effects on feed intake and weight gain. Additionally, goats have a higher ability to adapt to condensed tannin-containing forage or diet, and when condensed tannin is present at low or medium levels (<50 g/kg DM), condensed tannin may benefit goats without affecting feed intake and nutrient digestion [15].

The current study demonstrated an increase in CP digestibility, with no effect on DM, NDF, and ADF digestibility, after the inclusion of hydrolyzable tannin rather than condensed tannin in the diet of goats at a level of 0.5%. An explanation may be that compared to condensed tannin, hydrolyzable tannin had a lower binding affinity to dietary protein, leading to a greater proportion of dietary protein to produce ammonia N by rumen microbial degradation [12]. An increased amounts of rumen ammonia N may be absorbed into the blood and result in a higher N excretion in urine, not in feces, thus contributing to an increased apparent CP digestibility.

The amount of N intake by the goats was similar across groups, which could be explained by the lack of differences in total DM and CP intake among groups. However, dietary supplementation with hydrolyzable tannin (e.g., CT) and condensed tannin (e.g., QT) decreased fecal N output and total N output, indicating that both types of tannins can reduce N loss in goats. Additionally, this study found that goats fed a condensed tannin diet (e.g., QT) had lower urine N output than those fed the control diet, suggesting that condensed tannin may modulate N metabolism. Although N intake and fecal N output were similar in tannin-supplemented groups, condensed tannin numerically decreased urine N output, leading to improved N retention and N utilization in goats. Similarly, Zhang et al [29] reported that feeding dairy cows a 3% condensed tannin diet reduced urine N output and increased N retention. Fonseca et al [7] also found that beef cattle fed 3%–6% condensed tannin diets had higher N retention than those fed a control diet. These findings suggested that supplementing goats with condensed tannin may enhance N retention by reducing fecal and urine N output, thereby improving N utilization efficiency.

Urea is the dominant nitrogenous compound in ruminant urine, accounting for 52% to 94% of total urine N [30]. The present study showed that supplementing diets with 0.5% tannins, particularly condensed tannin, did not affect urine urea excretion, suggesting that this supplementation level was insufficient to influence urea output. In ruminants, allantoin, uric acid, and creatinine represent other major urinary nitrogenous compounds. Allantoin and uric acid are end products of purine metabolism, and their urine concentrations correlate with ruminal nucleic acid concentrations, thereby indirectly reflecting microbial N production [31]. In goats, urine allantoin excretion shows a positive relationship with digestible organic matter intake [32]. The observed increase in urine allantoin concentration in goats fed condensed tannin may be attributable to their enhanced feed intake. However, Johnson et al [33] reported that while microbial N flow increased, urine allantoin excretion decreased, suggesting that uric acid excretion may be more reliable indicator of microbial N flow than allantoin. The current study found that tannin supplementation reduced urine uric acid excretion, indicating decreased rumen microbial N flow to the small intestine. Condensed tannin produced a more pronounced reduction in urine uric acid concentration than hydrolyzable tannin, suggesting stronger microbial inhibition potential. Creatinine, a byproduct of energy metabolism in muscles, shows positive correlation with BW and serves as a urine volume marker [34]. The decreased urine creatinine excretion observed in goats fed hydrolyzable tannin suggests reduced muscle metabolism and protein retention compared to condensed tannin, consistent with the finding that hydrolyzable tannin resulted in lower ADG than condensed tannin.

The addition of tannins to the diet alters rumen fermentation pattern, as observed in this study. TA, a weak acid containing carboxy groups (-COOH), naturally lowers rumen pH. Consistent with this, goats fed TA diet exhibited reduced rumen pH. Tannin can form complexes with proteins and carbohydrates, inhibiting extracellular enzymes and limiting substrate availability for microbial degradation, thereby reducing NH_3_-N and VFAs production [35]. However, in the present study, VFA concentrations remained unchanged, while rumen NH_3_-N concentration increased in goats fed both hydrolyzable and condensed tannins diets. The inclusion of tannin increased the NH_3_-N concentration of the rumen. This effect may be due to stimulating the activity of protein-degrading bacteria, but the exact mechanism of action should be studied further. As both predators and symbionts, protozoa modulate rumen microbiota composition and dynamics. However, protozoa negatively affect N utilization efficiency by preying on microbes and degrading MCPs, promoting intraruminal N recycling [36]. In this study, tannin-supplemented diets reduced protozoa populations but increased NH_3_-N concentrations and N utilization, suggesting that tannins may influence N metabolism by regulating protozoa activity in goats.

In the current study, supplementation of hydrolyzable tannin and condensed tannin elevated plasma levels of total protein, albumin, and globulin. Albumin, primarily synthesized in the liver, increased in tannin-fed goats, suggesting enhanced protein synthesis [37]. This aligns with the observed improvements in ADG and N retention. Elevated globulin levels in tannin groups, along with IgA in the condensed tannin group, indicate potential immune-enhancing effects of tannins. Similarly, Tian et al [13] reported that dietary inclusion of 0.4% condensed tannin boosted serum IgA concentration in Sichuan black goats. Excessive reactive oxygen species (ROS) accumulation can induce oxidation stress in ruminants. SOD and CAT, critical antioxidant enzymes, neutralize ROS into less harmful compounds, mitigating oxidative damage to biomolecules. MDA, a lipid peroxidation byproduct, serves as a key oxidative stress biomarker [38]. This study demonstrated that condensed tannin (e.g., QT) supplementation increased plasma SOD, CAT, and T-AOC activities while reducing MDA levels, underscoring the antioxidant potential of tannins, particularly condensed tannin, in goats. The explanations for the enhancement of antioxidant capacity of tannins may be that tannins act as exogenous antioxidants either being degraded and absorbed into the bloodstream and/or removing or chelating prooxidant compounds in the lumen of the gastrointestinal tract, due to the strong reducibility of the phenolic hydroxyl groups of tannins [9]. Furthermore, compared to hydrolyzable tannin, condensed tannin has more hydroxyl groups, which can be more easily oxidized, thus having higher antioxidant activity.

## CONCLUSION

This study demonstrated that effects of tannin on growth performance, N metabolism, rumen fermentation, and blood parameters varied by tannin type. QT increased ADFI and ADG compared to TA, suggesting growth-promoting effects of condensed tannin. While tannin supplementation did not significantly affect nutrient digestibility, QT decreased fecal N and urine N output while improving N retention and utilization relative to CT and TA. These results indicate that condensed tannin enhance N balance more effectively than hydrolyzable tannin at equivalent dietary inclusion levels. Both hydrolyzable tannin and condensed tannin increased rumen NH_3_-N concentration and reduced protozoa counts, demonstrating their rumen fermentation-modulating properties. Goats fed QT diets showed elevated plasma SOD, CAT, and IgA levels, confirming condensed tannin’s antioxidant and immune-enhancing potential. Given these positive impacts on growth performance, N metabolism, antioxidant status, and immune regulation, future studies should investigate the biological mechanisms of condensed tannin in Liaoning cashmere goats to optimize their dietary application.

## Figures and Tables

**Table 1:** Ingredients and nutritional level of the basal diet (DM basis)

Items	Contents	Items	Contents
Ingredients	Level (%)	Nutrients^1)^	Level (%)
Alfalfa	40.00	DM	89.43
Peanut straw	35.00	DE (MJ/kg)	9.83
Corn	13.25	CP	14.25
Soybean meal	6.25	NDF	42.72
Rapeseed meal	0.50	ADF	29.24
Corn gluten meal	1.00	Ca	1.00
DDGS	1.75	TP	0.46
Liquid molasses	0.75	Ash	8.52
Limestone	0.50	EE	3.91
Bentonite	0.25		
Premix^2)^	0.30		
Sodium bicarbonate	0.25		
Sodium chloride	0.20		
Total	100.00		

1) DE value was calculated, while other values were measured.

2) Per kg diet provides: Fe, 21,300 mg; Cu, 18,600 mg; Mn, 16,000 mg; Zn, 58,600 mg; I, 900 mg; Se, 350 mg; Co, 270 mg; VA, 2,000,000 IU; VD_3_, 6,000,000 IU; VE, 40,000 mg;

DM, dry matter; DE, digestible energy; CP, crude protein; NDF, neutral detergent fiber; ADF, acid detergent fiber; Ca, calcium; TP, total phosphorus; EE, ether extract.

**Table 2:** Effects of different tannins on growth performance of Liaoning cashmere goats

Items	Groups				p-value

	Control	CT	TA	QT	
BW (kg)					
1 d^1)^	32.36±5.56	32.70±6.55	32.84±8.10	31.78±6.07	0.471
21 d	34.01±5.53	34.51±6.51	34.60±8.15	33.75±5.62	0.590
42 d^1)^	35.91±5.51	36.46±6.55	36.61±8.31	35.87±5.47	0.590
ADG (g)					
1–21 d	78.30^b^±7.06	86.42^ab^±10.29	77.72^b^±77.91	97.08^a^±23.98	0.024
22–42 d	90.57^b^±11.66	92.75^ab^±7.65	93.06^b^±13.28	100.59^a^±14.08	0.026
1–42 d	84.44^b^±7.20	89.50^ab^±8.71	81.86^b^±12.47	100.73^a^±16.41	0.009
ADFI (g)					
1–21 d	957^b^±19.42	956^b^±23.25	897^c^±23.76	984^a^±24.12	<0.01
22–42 d	1,022^b^±28.22	1,027^b^±30.88	978^c^±32.49	1,134^a^±31.45	<0.01
1–42 d^1)^	989^b^±40.86	992^b^±44.93	908^c^±30.12	1,031^a^±36.58	<0.01
F/G (g/g)					
1–21 d	12.32±1.34	9.96±0.83	11.90±2.65	11.63±2.45	0.074
22–42 d	11.43±1.31	10.46±0.93	10.76±2.40	12.14±2.56	0.276
1–42 d^1)^	11.79±1.00	10.82±1.08	11.30±2.51	11.29±1.84	0.702

1) Values in the Control, TA, and QT groups are quoted from Zhu et al [20].

a–c Means in a row with different superscript letters differ significantly at p<0.05.

CT, chestnut tannin; TA, tannic acid; QT, quebracho tannin; BW, Body weight; ADG, average daily gain; ADFI, average daily feed intake; F/G, feed-to-gain ratio.

**Table 3:** Effects of different tannins on nutrient digestibility of Liaoning cashmere goats

Items	Groups				p-value

	Control	CT	TA	QT	
Nutrient intake (g/d)					
DM	1,037±61.51	1,012±58.10	1,016±99.30	1,031±68.00	0.870
CP	135±2.50	132±2.01	132±2.32	134±1.21	0.086
EE	12±3.48	11±2.44	14±7.61	15±5.08	0.345
NDF	469±42.45	488±80.90	440±65.56	427±43.39	0.156
ADF	274^ab^±30.90	285^a^±71.62	268^ab^±64.46	244^b^±27.61	<0.01
Nutrient digestibility (g/kg)					
DM^1)^	822±34.80	811±52.10	801±53.40	816±20.20	0.939
CP^1)^	718^b^±41.10	762^a^±24.30	759^a^±74.10	723^b^±21.70	0.139
EE	600±11.58	558±14.42	619±19.27	627±13.20	0.757
NDF^1)^	689±60.20	728±52.70	644±50.30	638±59.30	0.144
ADF^1)^	565±68.10	574±108.20	534±83.30	515±55.70	0.929

1) Values in the Control, TA, and QT groups are quoted from Zhu et al [20].

a,b Means in a row with different superscript letters differ significantly at p<0.05.

CT, chestnut tannin; TA, tannic acid; QT, quebracho tannin; DM, dry matter; CP, crude protein; EE, ether extract; NDF, neutral detergent fiber; ADF, acid detergent fiber.

**Table 4:** Effects of different tannins on N metabolism of Liaoning cashmere goats

Items	Groups				p-value

	Control	CT	TA	QT	
N intake (g/d)	21.8±0.43	21.1±0.65	21.1±0.58	21.5±0.38	0.454
Fecal N output (g/d)	7.7^a^±0.94	6.6^b^±0.91	6.6^b^±1.01	6.5^b^±0.42	0.047
Urine N output (g/d)	5.6^a^±0.68	5.1^ab^±1.02	5.4^ab^±1.13	4.5^b^±1.11	0.038
Total N output (g/d)	13.3^a^±1.38	11.6^b^±1.62	12.0^ab^±1.33	11.0^b^±1.00	0.025
Fecal N output/total N output (g/g)	0.57±0.10	0.53±0.05	0.55±0.11	0.58±0.11	0.252
Urine N output/total N output (g/g)	0.42±0.10	0.46±0.05	0.44±0.11	0.41±0.07	0.106
Digestible N (g/d)	13.9^b^±0.53	14.5^a^±1.18	14.5^a^±1.27	15.0^a^±1.03	0.023
N retention (g/d)	8.2^c^±0.51	9.4^b^±1.53	9.1^b^±1.31	10.4^a^±1.05	<0.01
N utilization (g/kg)	382^c^±71.7	421^b^±14.7	431^b^±39.1	486^a^±75.6	0.012

a–c Means in a row with different superscript letters differ significantly at p<0.05.

CT, chestnut tannin; TA, tannic acid; QT, quebracho tannin.

**Table 5:** Effects of different tannins on urine N constituents of Liaoning cashmere goats

Items	Groups				p-value

	Control	CT	TA	QT	
Urea (mmol/d)	148±20.56	146±18.80	144±21.36	143±18.88	0.681
Allantoin (mmol/d)	35.6^b^±3.78	35.0^b^±4.33	47.8^a^±7.50	48.1^a^±7.29	<0.01
Uric acid (mmol/d)	2.9^a^±0.48	2.7^b^±0.45	2.7^b^±0.58	2.5^c^±0.49	0.029
Creatinine (mmol/d)	3.2^a^±1.23	2.6^b^±1.18	2.6^b^±1.24	3.6^a^±1.82	0.023

a–c Means in a row with different superscript letters differ significantly at p<0.05.

CT, chestnut tannin; TA, tannic acid; QT, quebracho tannin.

**Table 6:** Effects of different tannins on rumen fermentation parameters of Liaoning cashmere goats

Items	Groups				p-value

	Control	CT	TA	QT	
pH^1)^	6.81^a^±0.13	6.75^a^±0.23	6.42^b^±0.33	6.74^a^±0.15	0.004
NH_3_-N (mg/dL)^1)^	7.1^b^±0.31	9.5^a^±0.09	10.1^a^±0.08	9.6^a^±0.16	0.007
Protozoa number (10^5^/mL)	4.90^a^±0.50	3.83^b^±0.78	3.18^b^±0.83	3.35^b^±0.59	<0.01
MCP (g/L)	4.97±0.52	4.22±0.52	4.74±0.52	4.73±0.52	0.583
Total VFAs (mmol/L)^1)^	74.69±20.91	71.36±15.74	70.78±21.18	65.84±12.58	0.409
Acetate (mmol/L)	49.47±13.46	47.35±10.55	47.71±13.68	44.25±8.33	0.141
Propionate (mmol/L)	13.06±4.42	11.24±2.71	11.74±4.78	9.97±2.87	0.253
Butyrate (mmol/L)	9.41±3.01	9.49±3.24	8.51±2.31	8.44±2.42	0.143
Isobutyrate (mmol/L)	1.63^ab^±0.51	2.25^a^±0.92	1.35^b^±0.65	1.57^ab^±0.55	0.045
Valerate (mmol/L)	0.55±0.19	0.49±0.08	0.71±0.36	0.42±0.14	0.128
Isovalerate (mmol/L)	0.56±0.14	0.52±0.28	0.75±0.41	1.19±1.58	0.174
Acetate/propionate	3.91±0.67	4.27±0.87	4.27±1.03	4.57±0.87	0.210

1) Values in the Control, TA, and QT groups are quoted from Zhu et al [20].

a,b Means in a row with different superscript letters differ significantly at p<0.05.

CT, chestnut tannin; TA, tannic acid; QT, quebracho tannin; NH_3_-N, ammonia-N; MCP, microbial protein; VFA, volatile fatty acid.

**Table 7:** Effects of different tannins on plasma biochemical indicators of Liaoning cashmere goats

Items	Groups				p-value

	Control	CT	TA	QT	
Total protein (g/L)	70.59^c^±5.80	78.37^b^±3.78	80.60^b^±6.51	85.76^a^±3.75	<0.01
Albumin (g/L)	25.48^b^±2.61	26.48^ab^±3.15	28.32^a^±1.01	28.35^a^±1.39	0.022
Globulin (g/L)	45.11^c^±5.55	51.88^b^±4.02	52.27^b^±6.68	57.44^a^±3.13	<0.01
Urea N (mmol/L)	8.93±1.24	8.78±1.37	8.22±2.64	7.74±1.70	0.498
Uric acid (μmol/L)	2.72±1.10	2.13±1.05	2.39±1.45	2.47±0.91	0.753
AST (U/L)	66.69±8.68	65.28±9.65	68.81±7.73	76.72±10.88	0.061
ALT (U/L)	23.71±3.78	21.06±5.37	31.86±4.59	23.02±4.67	0.318
Total cholesterol (mmol/L)	1.74^b^±0.18	1.87^b^±0.41	2.25^a^±0.36	2.24^a^±0.17	0.002
Triglyceride (mmol/L)	0.34±0.16	0.39±0.11	0.25±0.08	0.32±0.13	0.190
Glucose (mmol/L)	3.32±0.85	3.04±0.85	4.27±2.17	3.33±0.67	0.218

a–c Means in a row with different superscript letters differ significantly at p<0.05.

CT, chestnut tannin; TA, tannic acid; QT, quebracho tannin; AST, aspertate aminotransferase; ALT, alanine transaminase.

**Table 8:** Effects of different tannins on plasma antioxidant indicators of Liaoning cashmere goats

Items	Groups				p-value

	Control	CT	TA	QT	
SOD (U/mL)	84.87^b^±9.61	74.33^bc^±12.66	69.86^c^±15.55	103.64^a^±16.04	<0.01
CAT (U/mL)	45.55^b^±4.17	39.92^c^±3.58	36.78^c^±2.73	51.81^a^±2.41	<0.01
ALP (U/L)	235.02±61.24	232.33±33.34	218.59±33.20	222.99±37.03	0.830
LDH (U/L)	168.95±20.45	168.04±49.91	226.56±19.00	207.96±43.75	0.206
MDA (μmol/L)	3.26^a^±0.38	2.26^b^±0.32	1.38^c^±0.28	1.82^b^±0.22	<0.01
T-AOC (U/mL)	12.52^b^±0.80	13.43^b^±0.91	14.61^a^±1.19	14.43^a^±1.06	<0.01

a–c Means in a row with different superscript letters differ significantly at p<0.05.

CT, chestnut tannin; TA, tannic acid; QT, quebracho tannin; SOD, superoxide dismutase; CAT, catalase; ALP, alkaline phosphatase; LDH, lactic dehydrogenase; MDA, malonaldehyde; T-AOC, total antioxidant capacity.

**Table 9:** Effects of different tannins on plasma immune indicators of Liaoning cashmere goats

Items	Groups				p-value

	Control	CT	TA	QT	
IgA (g/L)	1.57^b^±0.18	1.66^ab^±0.17	1.60^b^±0.16	1.80^a^±0.13	0.028
IgG (g/L)	19.29±1.00	20.98±3.94	20.36±1.49	21.25±1.88	0.316
IgM (g/L)	1.35±0.19	1.48±0.24	1.37±0.23	1.56±0.11	0.110
Lysozyme (U/mL)	304^c^±41.98	364^b^±37.32	347^b^±46.64	434^a^±19.48	<0.01

a–c Means in a row with different superscript letters differ significantly at p<0.05.

CT, chestnut tannin; TA, tannic acid; QT, quebracho tannin; IgA, immune globulin A; IgG, immune globulin G; IgM, immune globulin M.
